# Surface Tension of Infinitely Planar Surfaces from Nucleation Free Energies: A Comparison of Monte Carlo Calculations and Classical Theories

**DOI:** 10.1021/acs.jctc.5c01122

**Published:** 2025-08-18

**Authors:** Bin Chen, Ngoc My Nhi Nguyen

**Affiliations:** † Department of Chemistry, 5779 Louisiana State University, Baton Rouge, Louisiana 70803-1804, United States; ‡ Cain Department of Chemical Engineering, 5779 Louisiana State University, Baton Rouge, Louisiana 70803-1804, United States

## Abstract

A new approach for calculating the surface tension of an infinitely planar surface is presented, based on the thermodynamic relationship between clusters of different sizes. This method utilizes the aggregation-volume-bias Monte Carlo technique to compute the nucleation free energies of clusters of varying sizes. These free energy data are then compared with the predictions of conventional theories, such as classical nucleation theory and the Tolman equation, revealing that these theories accurately capture the size-dependency of the free energy for sufficiently large clusters. Key results from this analysis include the surface tension of an infinitely large cluster, corresponding to an infinitely planar surface, the Tolman length, and the chemical potential of the phases in equilibrium. The method is applied to two systems—Lennard–Jones and TIP4P/2005 water—and the calculated surface tension values for the infinitely large system (*γ* ^∞^) show excellent agreement with results from other established approaches.

## Graphical abstract

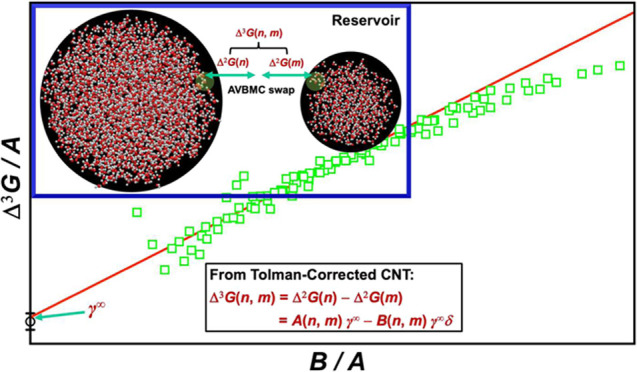

## 1 Introduction

Surface tension is the most important thermodynamic property when describing systems with surfaces and interfaces, such as clusters, and is key to understanding many important processes and phenomena from phase transition to biological self-assembly.^1–3^ Surface tension arises from the imbalance of intermolecular forces experienced by molecules at the surface compared to those in the bulk. Molecules at the surface are subjected to attractive forces from interior and laterally, but not from exterior, resulting in a net inward force that acts to minimize the surface area of the bulk. In systems with well-defined surfaces, such as liquid droplets or thin films, surface tension plays a crucial role in determining the shape and stability of these structures. It is typically quantified as the force per unit length or the energy per unit area required to stretch the surface.

Various simulation methods based on either the mechanic or thermodynamic definition of surface tension have been developed to compute surface tension using atomistic models. The former, which is more commonly used, involves calculating pressure tensors, often via the molecular virial.^4–10^ In contrast, the thermodynamic approach calculates the change in free energy with respect to a change in surface area.^11–14^ In addition to these mechanic and thermodynamic routes, one can also determine surface tension from the free energy barrier between vapor and liquid phase.^15–17^

Although many methods are available, surface tension remains as one of the most difficult thermodynamic properties to compute with precision. Even for the simplest Lennard–Jones system, the surface tension data reported by various groups exhibit a significantly larger spread (±4%) compared to other properties, such as saturated liquid density (±0.2%).^18^ This discrepancy arises because surface tension is much more sensitive to factors such as the truncation of intermolecular interactions or the treatment of long-range corrections. Additionally, the calculated surface tension is expected to be dependent on system size. To obtain the infinite-system-size surface tension, calculations are performed at various system sizes, and the resulting data are used to extrapolate the surface tension for an infinite system.^19–21^

In this work, a cluster-based approach is presented to calculate surface tension. This approach is motivated by previous findings showing that the size dependence of the nucleation free energy of clusters can be used to extrapolate bulk-phase properties, including surface tension.^22–27^ The aggregation-volume-bias Monte Carlo technique^28,29^ is employed to compute the nucleation free energy over a wide range of cluster sizes for two systems: Lennard–Jones and TIP4P/2005 ^30^ water. Comparison with theoretical predictions reveals that accurately capturing the size dependence of nucleation free energy requires combining classical nucleation theory^31–36^ with the Tolman equation^37^ to properly account for the thermodynamics of curved interfaces. A key outcome of this analysis is the surface tension of an infinitely planar surface.

## 2 Methods

This cluster-based approach to surface tension calculation hinges on the synergism of the availability of an efficient computational method—aggregation-volume-bias Monte Carlo—to obtain the nucleation free energies (NFEs) for clusters of various sizes, and the ability to interpolate and extrapolate these NFEs across the full size range, including the infinite limit corresponding to the bulk phase, by leveraging their size dependence under the guidance of the classical theories.

The aggregation-volume-bias Monte Carlo (AVBMC) method^28,29^ was specifically developed for nucleation systems, as it enables efficient sampling of clusters of various sizes through particle swap moves, like those used in Gibbs ensemble Monte Carlo^38–40^ for studying bulk phase equilibria. A distinctive feature of AVBMC is its use of a local sampling volume, *V*_in_, typically a sphere centered on a target molecule, to facilitate these particle exchanges. In the original formulation,^28,29^ the target molecule is randomly selected from within the cluster. However, in large clusters, this approach tends to favor interior molecules—simply because they outnumber surface molecules—thereby reducing the acceptance rate of swap moves. To address this limitation, a preferential selection scheme has been introduced to bias the choice of the target molecule toward the cluster interface.^41^

Specifically, the target molecule is chosen using an energy-biased scheme. For particle insertion, a candidate molecule *k*, whose interaction energy with the rest of the cluster is *ε*_*k*_, is chosen as the target with probability

1 $$P_{\mathrm{target},\mathrm{ins}}^{\left(k\right)}=\frac{e^{\alpha \varepsilon_{k}}}{\sum_{i=1}^{n}e^{\alpha \varepsilon_{i}}}$$

where *α* is a positive biasing parameter and *n* is the cluster size. This formulation preferentially selects particles with higher interaction energies—typically located near the cluster surface—thus increasing the likelihood of targeting interfacial molecules for insertion moves. For particle removal, a candidate molecule is first selected as the target based on the interaction energy of its highest-energy neighbor. Specifically, the target molecule *k* is chosen with probability

2 $$P_{\mathrm{target},\mathrm{rem}}^{\left(k\right)}=\frac{e^{\beta \varepsilon_{k}^{\prime }}}{\sum_{i=1}^{n}e^{\beta \varepsilon_{i}^{\prime }}}$$

where *ε*_*k*_^′^ is the energy of the highest-energy neighbor of molecule *k*, and *β* is the inverse temperature. Once the target molecule is selected, one of its neighbors—say, particle *j*—is chosen for trial removal according to the probability

3 $$P_{\mathrm{select},\mathrm{rem}}^{\left(j\right)}=\frac{e^{\beta \varepsilon_{j}}}{\sum_{i=1}^{n\prime }e^{\beta \varepsilon_{i}}}$$

where *ε*_*j*_ is the interaction energy of neighbor *j*, and *n*′ denotes the number of neighbors of the target molecule. This approach ensures that particles with higher interaction energies are more likely to be selected for removal, thereby improving the acceptance rate of such moves. The biases introduced through these selection schemes are accounted for in the final acceptance criterion to maintain detailed balance and ensure reversibility.

To further improve the acceptance rate, a multiple insertion scheme—commonly used in configurational-bias Monte Carlo^42–47^ —is employed. In this approach, several trial configurations are generated, increasing the likelihood of finding an acceptable one. Selection among them is performed using the Rosenbluth weighting scheme^48^ as follows

4 $$w^{\left(m\right)}=\frac{e^{-\beta \varepsilon_{m}^{\mathrm{cut}}}}{W}$$

where the normalization factor *W* is given by

5 $$W=\sum_{i=1}^{n\prime \prime }e^{-\beta \varepsilon_{i}^{\mathrm{cut}}}$$

where *ε*_*i*_^cut^ is the interaction energy between trial configuration *i* and the surrounding molecules within a specific cutoff distance, and *n*″ denotes the number of trial configurations. For the trial configuration selected in the proposed insertion move—or the original (old) configuration in a deletion move—the interaction energy with the rest of the cluster, denoted *ε* ^tail^, is also computed and included in the final Metropolis acceptance criterion.^49^ In a grand canonical ensemble, where the cluster exchanges particles with a reservoir at chemical potential *μ*, the acceptance probabilities are given as follows.

- For insertion
6 $$P_{\mathrm{accept},\mathrm{ins}}=\min\left\{1,\frac{P_{\mathrm{target},\mathrm{rem}}\cdot P_{\mathrm{select},\mathrm{rem}}\cdot V_{\mathrm{in}}}{P_{\mathrm{target},\mathrm{ins}}}\cdot \frac{W}{n^{\prime \prime }}\cdot e^{\beta \mu -\beta \varepsilon^{\mathrm{tail}}}\right\}$$
- For deletion
7 $$P_{\mathrm{accept},\mathrm{rem}}=\min\left\{1,\frac{P_{\mathrm{target},\mathrm{ins}}}{P_{\mathrm{target},\mathrm{rem}}\cdot P_{\mathrm{select},\mathrm{rem}}\cdot V_{\mathrm{in}}}\cdot \frac{n^{\prime \prime }}{W}\cdot e^{-\beta \mu +\beta \varepsilon^{\mathrm{tail}}}\right\}$$
  Here, *V*_in_ is the insertion volume, *W* is the Rosenbluth normalization factor from eq 5, and all probability terms are defined as in the preceding equations. These acceptance criteria ensure detailed balance in the grand canonical ensemble while accounting for energy and biasing terms introduced during the selection process.

The umbrella sampling method^50^ was employed in all simulations to ensure uniform sampling of clusters of different sizes, thereby enabling consistent interpretation of nucleation free energies (NFEs) from cluster populations. An iterative procedure was used to converge the biasing potential required for effective sampling. Once the populations of different cluster sizes are similar or within a few percent from each other, production runs were performed in which each cluster size was sampled at least 10^10^ times. To accelerate this process, 20 to 80 independent simulations were performed using distinct initial configurations and random seeds. These simulations were grouped into five blocks, each containing multiple runs. The uncertainty was estimated from the standard deviation of the block averages.

All intermolecular interactions were fully included. The insertion volume, *V*_in_, was defined as a spherical region centered on the target molecule, with a radius of 1.5*σ* for the Lennard–Jones systems and 5 Å for TIP4P/2005 water (centered on the oxygen site). For particle insertion, an energy-biasing parameter *α* = 0.1*β* was used to preferentially target interfacial molecules during selection of the insertion site, based on prior experience.^41^ The number of trial configurations, *n*″, was set to 10 in all simulations.

A Stillinger cluster criterion^51^ was applied in all simulations: two particles were considered part of the same cluster if one lay within the insertion volume of the other. In addition to AVBMC swap moves, translational moves (and rotational moves for TIP4P/2005 water) were also employed, with equal probability assigned to each move type. For both particle deletion and translational moves, any proposed move that resulted in a violation of the cluster criterion was immediately rejected. Rotational moves for the water model were performed by rotating the molecule about its oxygen site.

Free energy calculations were performed sparsely but systematically over a broad range of cluster sizes, from 20 ± 2 to 8000 ± 2 particles. This range is sufficient to quantitatively evaluate the size dependence of NFEs, enabling both interpolation and extrapolation to estimate NFEs for all cluster sizes—including the infinite limit, which corresponds to the bulk phase. In parallel, extensive comparisons were made to classical theories to assess their ability to capture the observed size dependence. The results for the Lennard–Jones system were reported in reduced units unless noted otherwise.

## 3 Results and Discussion

Figure 1 shows the acceptance rate of particle swap moves as a function of cluster size for TIP4P/2005 water at 300 K. With the original AVBMC scheme—where the target particle is selected randomly from the cluster—the acceptance rate decreases as the cluster size increases, making convergence in NFE more challenging for larger clusters. However, using a preferential selection scheme biased toward the interfacial region significantly improves the acceptance rate across all cluster sizes, with a more than 100-fold increase at a cluster size of 8000 (see Table S1). Specifically, the acceptance rate rises from around 8% at a cluster size of 60 to over 10% at a size of 2000. A substantial portion of this improvement can be attributed to the part of the energy-based selection scheme to choose both the target and the candidate for particle removal, so that particles with higher interaction energies are preferentially selected for being removed from the cluster (see Figure 1 with *α* = 0). Further gains are observed when preferential selection of the interfacial region is employed for insertion (see Figure 1 with *α* = 0.1*β*), particularly for larger clusters.

**Figure 1:**
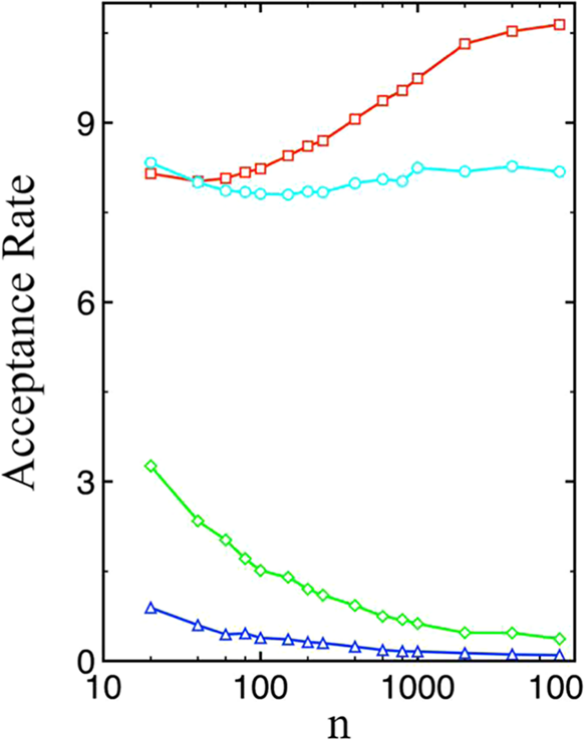
Acceptance rate (in %) as a function of cluster size, obtained using three selection schemes: preferential selection of both the target molecule and the molecule for removal with *α* = 0.1*β* (red squares) or *α* = 0 (cyan circles), preferential selection of the molecule for removal only (green diamonds), and random selection of both the target and removal molecules (blue triangles).

The size dependence of NFE, or Δ*G*, is often analyzed in its differential form using the so-called Δ^2^ *G* (or ΔΔ*G*) plots.^22–27,52–56^ These plots are particularly useful for disentangling the bulk and surface contributions to Δ*G*, due to their distinct dependencies on cluster size. According to the classical nucleation theory (CNT),^31–36^ for a perfectly spherical liquid cluster of radius *R* containing *n* particles under supersaturated conditions, the free energy is given by

8 $$\Delta G\left(n\right)=n\Delta \mu +4\pi R^{2}\gamma =n\Delta \mu +n^{2/3}{\left(\frac{36\pi }{\rho^{2}}\right)}^{1/3}\gamma$$

where Δ*μ* is the chemical potential difference between the supersaturated vapor and the stable liquid phase, *γ* is the surface tension (typically taken as the planar limit *γ* ^∞^), and *ρ* is the density (assumed equal to that of the liquid phase at coexistence). The first term represents the bulk contribution, while the second corresponds to the surface contribution, which scales with *n* ^2/3^. One finite difference form of Δ*G*(*n*) that has recently been introduced is given by Δ*G*(*n* + 2) – Δ*G*(*n* – 2).^27^ Based on CNT, or eq 8

9 $$\Delta^{2}G=\Delta G\left(n+2\right)-\Delta G\left(n-2\right)=4\Delta \mu +\left[{\left(n+2\right)}^{2/3}-{\left(n-2\right)}^{2/3}\right]{\left(\frac{36\pi }{\rho^{2}}\right)}^{1/3}\gamma$$

Thus, CNT predicts a linear relationship between Δ^2^ *G* and the size-dependent term (*n* + 2)^2/3^ – (*n* – 2)^2/3^, providing a convenient way to extract surface tension from simulation data.

Before presenting the main results, we first compare Δ^2^ *G* values from four simulation strategies previously shown to produce different acceptance rates. Although these approaches satisfy the detailed balance condition, it is important to verify that any bias introduced in move proposals—and its correction in the acceptance rule—is properly implemented, ensuring that the outcomes are not systematically affected.

Figure 2 shows Δ^2^ *G* for selected cluster sizes in (i) the Lennard–Jones (LJ) system at *T* = 0.7, *ρ*_v_ = 2.5 × 10^–3^, and (ii) TIP4P/2005 water at *T* = 300, *ρ*_v_ = 1 × 10^–6^ molecule Å^–3^. For LJ, the curves are nearly indistinguishable, though there is a small but systematic offset—about an order of magnitude smaller than in water (see Table S2). This offset barely affects the slope or surface tension, explaining the nearly identical LJ surface tension values from both methods, but it does shift the intercept, which relates to Δ*μ*, the chemical-potential difference between supersaturated vapor and stable liquid.

**Figure 2:**
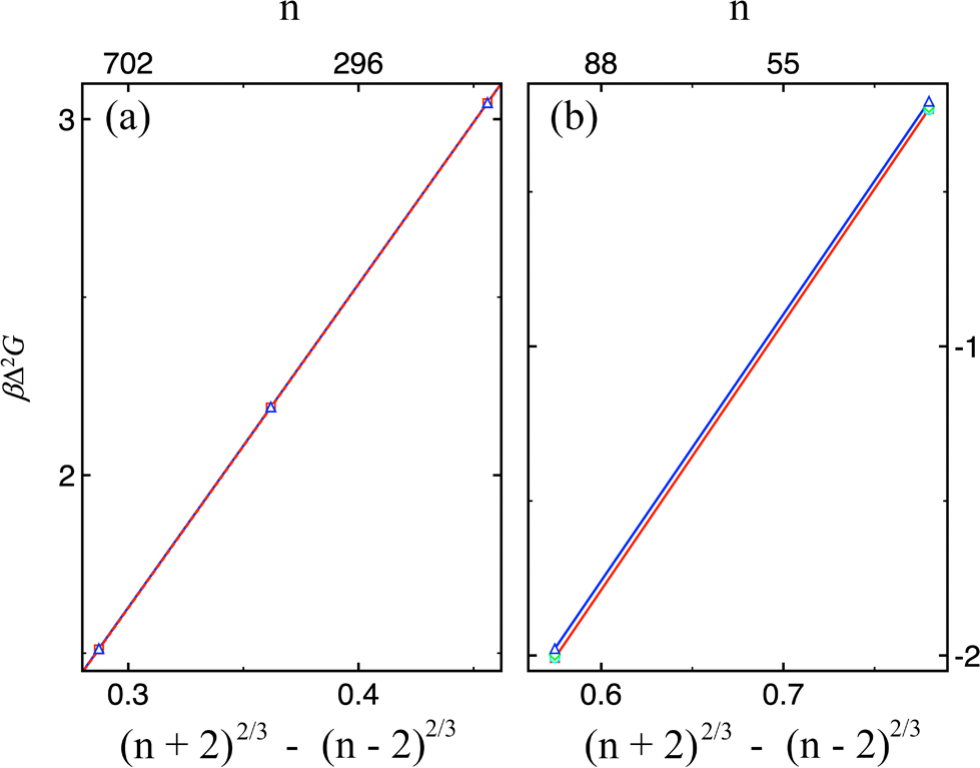
Comparison of Δ^2^ *G* values from four simulation strategies (symbols are the same as in Figure 1) for (a) Lennard–Jones (LJ) at *T* = 0.7, *ρ*_v_ = 2.5 × 10^–3^, and (b) TIP4P/2005 water at *T* = 300, *ρ*_v_ = 1 × 10^–6^ molecule Å^–3^. LJ results are nearly identical, apart from a slight systematic offset between preferential and fully random selection (see Table S2). For water, the offset is larger, with the random scheme consistently producing higher Δ^2^ *G*.

The method with preferential selection of both target and removal molecules yields a more negative Δ*μ* (by less than 0.01 *k*_B_ *T*), indicating better sampling of bulk liquid than the fully random scheme. Molecules with higher energies typically reside at the surface but can also occur within the liquid interior, where crowding hinders their relaxation via ordinary translational or rotational moves. Energy-biased selection can relocate these molecules to new environments, improving equilibration. On the contrary, in the fully random scheme, such molecules can remain “trapped” for disproportionately long times, much like clusters trapped in the gas phase—a limitation that motivates the development of advanced sampling methods such as AVBMC.^28^ This systematic shift is expected to increase with stronger intermolecular interactions (e.g., LJ to water) and with decreasing temperature.

Figure 3 presents the Δ^2^ *G* results for all cluster sizes investigated in both systems, allowing direct comparison of trends across the entire size range. For the Lennard–Jones system, the Δ^2^ *G* data follow a linear trend across most cluster sizes, as predicted by CNT, with deviations appearing for small clusters due to the breakdown of the capillary approximation—consistent with previous studies.^22–27^ By using a linear fit to the Δ^2^ *G* data for clusters in the size range from 200 to 800 and the slope value obtained, a surface tension of 1.1746 ± 0.0002 is estimated, which is in excellent agreement with the value of 1.18 ± 0.01 obtained from finite-size scaling and grand-canonical transition-matrix Monte Carlo simulations for an infinite system.^19^ In contrast, the water system exhibits a gradual change in slope with increasing cluster size, indicating a size-dependent surface tension arising from the curvature effect—another source of error in the original CNT, as most recently highlighted in a review.^57^

**Figure 3:**
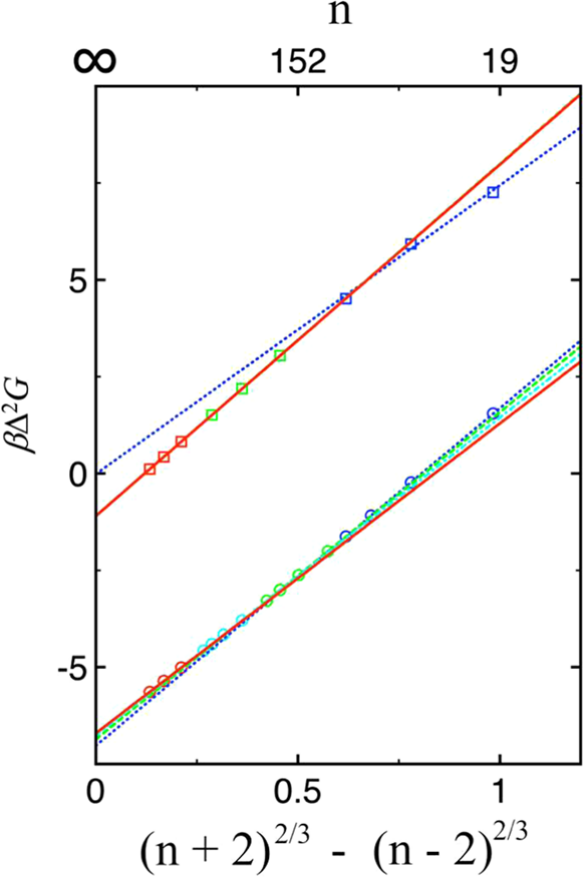
Δ^2^ *G* (= Δ*G*(*n* + 2) – Δ*G*(*n* – 2)) in units of *k*_B_ *T* as a function of (*n* + 2)^2/3^ – (*n* – 2)^2/3^ obtained for Lennard–Jones (squares) and TIP4P/2005 water (circles). Linear fits over different cluster size ranges are shown: blue dotted lines (20 to 80), green dashed lines (200 to 800 for LJ; 100 to 250 for water), cyan dash-dotted lines (400 to 1000 for water), and red solid lines (2000 to 8000).

The size dependence of surface tension for clusters has been a topic of interest since the early work of Tolman,^37^ who, building on the thermodynamic framework established by Gibbs,^58^ proposed the following relationship

10 $$\gamma \left(R\right)=\gamma^{\infty }\left(1-\frac{2\delta }{R}\right)$$

where *γ*(*R*) is the surface tension of a spherical interface with radius *R*, *γ* ^∞^ is the surface tension of an infinite, planar interface, and *δ* is the Tolman length, a parameter that characterizes the curvature dependence of surface tension.

To assess whether the size dependence of surface tension improves the theoretical prediction, the CNT expression for Δ*G*(*n*) is modified by including the Tolman correction, as follows

11 $$\Delta G\left(n\right)=n\Delta \mu +n^{2/3}{\left(\frac{36\pi }{\rho^{2}}\right)}^{1/3}\gamma^{\infty }\left(1-\frac{2\delta }{R}\right)$$

Since *R* ∝ *n* ^1/3^, the expression becomes

12 $$\Delta G\left(n\right)=n\Delta \mu +n^{2/3}{\left(\frac{36\pi }{\rho^{2}}\right)}^{1/3}\gamma^{\infty }-n^{1/3}{\left(\frac{384\pi^{2}}{\rho }\right)}^{1/3}\gamma^{\infty }\delta$$

From this, a finite-difference form of the second derivative becomes

13 $$\Delta^{2}G\left(n\right)=4\Delta \mu +\left[{\left(n+2\right)}^{2/3}-{\left(n-2\right)}^{2/3}\right]{\left(\frac{36\pi }{\rho^{2}}\right)}^{1/3}\gamma^{\infty }-\left[{\left(n+2\right)}^{1/3}-{\left(n-2\right)}^{1/3}\right]{\left(\frac{384\pi^{2}}{\rho }\right)}^{1/3}\gamma^{\infty }\delta$$

To analyze the curvature effect further, a third-order difference can be defined as

14 $$\Delta^{3}G\left(n,m\right)=\left[\Delta G\left(n+2\right)-\Delta G\left(n-2\right)\right]-\left[\Delta G\left(m+2\right)-\Delta G\left(m-2\right)\right]=a\left(n,m\right){\left(\frac{36\pi }{\rho^{2}}\right)}^{1/3}\gamma^{\infty }-b\left(n,m\right){\left(\frac{384\pi^{2}}{\rho }\right)}^{1/3}\gamma^{\infty }\delta$$

where

15 $$a\left(n,m\right)={\left(n+2\right)}^{2/3}-{\left(n-2\right)}^{2/3}-\left[{\left(m+2\right)}^{2/3}-{\left(m-2\right)}^{2/3}\right]$$

16 $$b\left(n,m\right)={\left(n+2\right)}^{1/3}-{\left(n-2\right)}^{1/3}-\left[{\left(m+2\right)}^{1/3}-{\left(m-2\right)}^{1/3}\right]$$

This expression allows one to fit simulation data to extract both *γ* ^∞^ and the Tolman length *δ*.

Figure 4 shows the Δ^3^ *G* results, scaled by *a*(*n*, *m*), for TIP4P/2005 water as a function of *b*(*n*, *m*)/*a*(*n*, *m*). Some data points exhibit large standard uncertainties, particularly those calculated from cluster pairs close in size (e.g., *n* = 1000 and *m* = 800) or involving large clusters. Despite this statistical noise, the results display a clear linear trend, except at small cluster sizes. A weighted linear fit to the data, excluding points with *n*, *m* ≤ 80, yields an intercept of 7.708 ± 0.028 *k*_B_ *T* and a slope of 3.92 ± 0.19 *k*_B_ *T*. Combined with the bulk liquid density of 0.9965 g/mL at 300 K,^30^ these values correspond to a planar surface tension *γ* ^∞^ = 68.4 ± 0.2 mN/m and a Tolman length *δ* = −0.49 ± 0.02 Å. The extrapolated *γ* ^∞^ agrees remarkably well with the previously reported value of 68.2 ± 0.3 mN/m obtained by Hu and Wang,^21^ who examined the surface tension’s dependence on the van der Waals cutoff radius *r*_vdW_ and extrapolated to the infinite cutoff limit. Similar surface tension results are obtained for this water model by Vega and de Miguel^59^ (69.3 ± 0.9 mN/m), and by Alejandre and Chapela^60^ (68.4 ± 1.1 mN/m). The weighted fit is used primarily to address the problem of closely spaced cluster sizes, which are common among midsized clusters and correspond to the largest uncertainties in Figure 4. Applying weights reduces the disproportionate influence of these densely sampled clusters, yielding a more balanced representation across all sizes.

**Figure 4:**
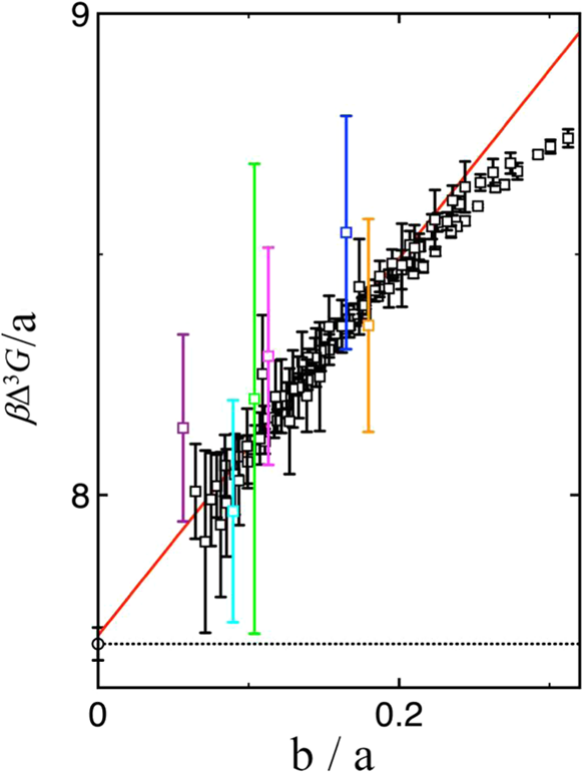
Δ^3^ *G*/*a*(*n*, *m*) in units of *k*_B_ *T* as a function of *b*(*n*, *m*)/*a*(*n*, *m*) obtained for TIP4P/2005 water (squares). A weighted linear fit to the data, excluding points with *n*, *m* ≤ 80, is shown as a red solid line. The intercept obtained from this linear fit corresponds to (36π/*ρ* ^2^)^1/3^ *γ* ^∞^ (see eq 14). The dotted black line and circle along the *y*-axis indicate the location of *γ* ^∞^, as determined by bulk-phase simulations,^21^ scaled by a factor of (36π/*ρ* ^2^)^1/3^. The pair *n* = 1000, *m* = 800 (highest uncertainty) is highlighted in green, followed by *n* = 250, *m* = 200 (second highest uncertainty) in blue. The third highest, *n* = 2000, *m* = 1000, is shown in cyan; the fourth, *n* = 800, *m* = 600, in magenta; the fifth, *n* = 200, *m* = 150, in orange; and the sixth, *n* = 8000, *m* = 4000, in purple.

Figure 5 shows how the extrapolated slope (i.e., *γ* ^∞^) depends on the range of cluster sizes included in the analysis. The figure clearly shows that small clusters—particularly those containing approximately 40 molecules or fewer—must be excluded from the extrapolation, as their inclusion leads to a noticeable overestimation of *γ* ^∞^. While increasing the range of cluster sizes reduces the uncertainty in the estimated *γ* ^∞^, it also significantly increases the computational cost. Accuracy comparable to that reported by Hu and Wang^21^ can be achieved using clusters up to 2000 molecules. Reasonable estimates of *γ* ^∞^ can still be obtained from smaller cluster ranges, although the statistical uncertainty increases—particularly due to the reliance on closely sized cluster pairs—when the simulation length is held constant.

**Figure 5:**
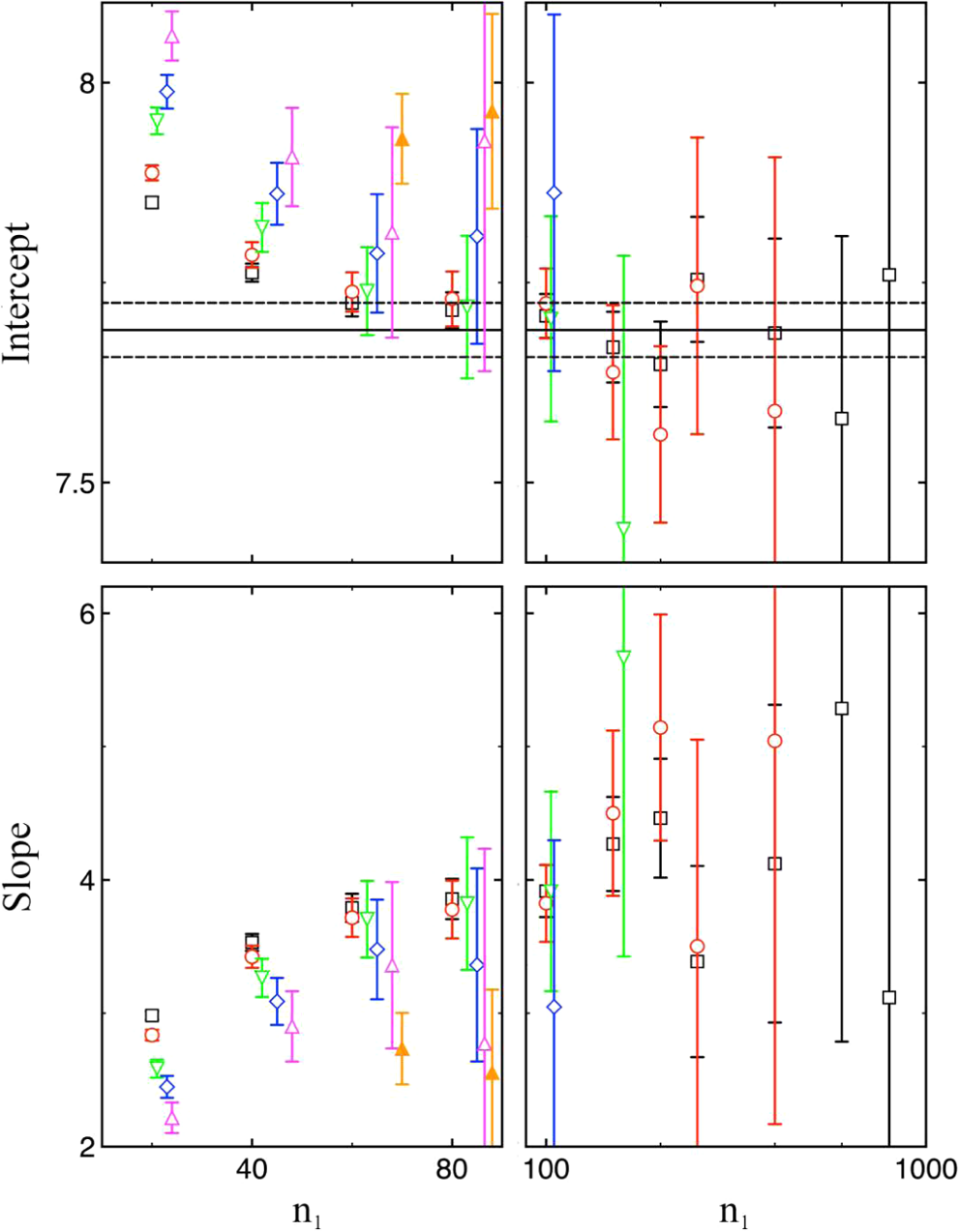
Intercept (top) and slope (bottom) values from weighted linear fits to the Δ^3^ *G* data in Figure 4 are shown for different cluster size ranges [*n*_1_, *n*_2_]. The values are plotted as a function of *n*_1_ using either a linear scale (left) or a logarithmic scale (right). Results obtained by sampling each cluster at least 10^10^ times are shown for *n*_2_ = 8000 (black squares), 2000 (red circles), 600 (green downward triangles), 400 (blue diamonds), and 250 (magenta upward triangles). Data corresponding to *n*_2_ = 600, 400, and 250 have been slightly shifted along the *x*-axis to improve visual clarity. Additional results obtained by sampling each cluster at least 10^11^ times for *n*_2_ = 250 are shown as filled orange upward triangles. The intercept and the slope obtained from this linear fit corresponds to (36π/*ρ* ^2^)^1/3^ *γ* ^∞^ and −(384π^2^/*ρ*)^1/3^ *γ* ^∞^ *δ*, respectively (see eq 14). The black line indicates the value of *γ* ^∞^, determined from bulk-phase simulations,^21^ scaled by (36π/*ρ* ^2^)^1/3^, while the two dashed lines represent the associated uncertainty reported in ref 21.

Similar conclusions can be drawn from this figure on how the extrapolated intercept (i.e., the Tolman length *δ*) depends on the range of cluster sizes included in the analysis. However, the associated uncertainties are noticeably larger than those for *γ* ^∞^.

There remains a lack of consensus in the literature regarding the Tolman length—its magnitude, and even its sign, are still debated.^61–65^ Nevertheless, the value of −0.49 ± 0.02 Å extrapolated from this simulation study by using a cluster size range between 100 and 8000 closely matches several previous estimates. For example, Leong and Wang^62^ applied the Young–Laplace equation^66–68^ to a different water model at 298 K and obtained a value of −0.48 Å. Using the mitosis method, Joswiak et al.^69^ determined *δ* = −0.56 ± 0.09 Å for TIP4P/2005 water at 300 K. Wilhelmsen et al.,^70^ employing classical density functional theory (c-DFT), estimated a Tolman length of −0.5 Å. Experimental work by Azouzi et al.,^71^ based on cavitation in quartz inclusions near 320 K, yielded a value of −0.47 Å.

Both *γ* ^∞^ and *δ* can also be extrapolated from a weighted fit to Δ^2^ *G* obtained at various cluster sizes using eq 13. The corresponding results are shown in Figure 6, which closely resemble those previously extrapolated via the linear fit to eq 14, as shown previously in Figure 5. It should be noted that eq 13 includes Δ*μ* explicitly in the fit (a three-parameter or planar fit), whereas eq 14 eliminates Δ*μ* and applies a linear fit to a higher-order difference. Because the two fits involve substantially different methodologies, they can be regarded as independent estimations; their agreement (or disagreement) provides an indication of the uncertainty. As shown in Table S9, the two sets of results agree within their respective uncertainties. For example, using the Δ^2^ *G* data within the cluster size range of 100 to 8000, *γ* ^∞^ and *δ* were determined to be 68.4 ± 0.03 mN/m and 0.48 ± 0.03 Å, respectively. Using an alternative cluster size range (80–8000), both eqs 13 and 14 yield a Tolman length of 0.48 ± 0.02 Å. Additionally, the chemical potential difference Δ*μ* can be obtained through this weighted fit. This quantity can be used to determine the supersaturation via the relation *S*_nucl_ = exp­(−*β*Δ*μ*). Using the Δ^2^ *G* data within the cluster size range of 100 to 8000, obtained at *T* = 300 K and *ρ*_v_ = 1 × 10^–6^ molecule/Å^3^, Δ*μ* was determined to be −1.670 ± 0.001 *k*_B_ *T*, corresponding to a *S*_nucl_ value of 5.312 ± 0.007. The saturated vapor density for this water model at 300 K is 5.642 ± 0.005 g/mL (1.885 × 10^–7^ molecule/Å^3^).^72^ Assuming ideal gas behavior, this gives a supersaturation ratio *S*_nucl_ of 5.302 when compared to our vapor density *ρ*_v_ = 1 × 10^–6^ molecule/Å^3^. Alternatively, using the pressure ratio definition of *S*_nucl_,^73^ the saturated vapor pressure at 300 K is 7.78 × 10^–3^ bar,^72^ while the supersaturated vapor pressure for an ideal gas at a vapor density *ρ*_v_ = 1 × 10^–6^ molecule/Å^3^ is 4.142 × 10^–2^ bar, yielding *S*_nucl_ = 5.324. Both approaches give nearly identical results, indicating that the vapor phase is close to ideal. Applying the ideal gas law to the saturated density gives a vapor pressure of 7.808 × 10^–3^ bar, in excellent agreement with the measured value. Among the three quantities extrapolated from the weighted fit, Δ*μ* exhibits the smallest uncertainty, while *δ* shows the largest—consistent with their contrasting scaling in Δ*G*: Δ*μ* contributes linearly (∝ *n*), whereas *δ* appears in a sublinear term (∝ *n* ^1/3^) (see eq 12).

**Figure 6:**
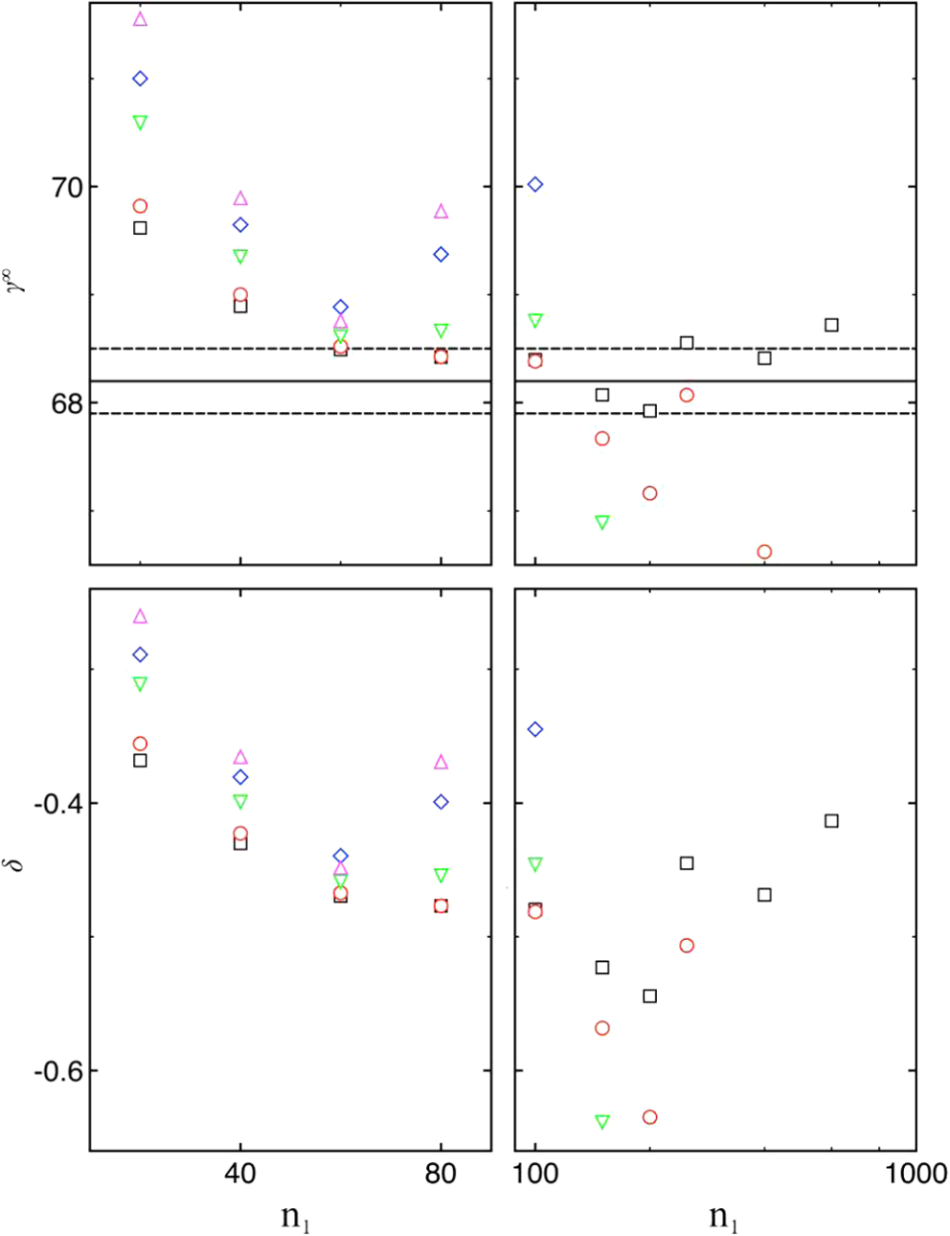
Surface tension in mN/m (top) and Tolman length in Å (bottom) obtained from weighted planar fits to the Δ^2^ *G* data using eq 13 over different cluster size ranges [*n*_1_, *n*_2_]. The values are plotted as a function of *n*_1_ using either a linear scale (left) or a logarithmic scale (right). Results are shown for *n*_2_ = 8000 (black squares), 2000 (red circles), 600 (green downward triangles), 400 (blue diamonds), and 250 (magenta upward triangles). The black line indicates the value of *γ* ^∞^, determined from bulk-phase simulations,^21^ while the two dashed lines represent the associated uncertainty reported in ref 21.

Direct measurements of surface tension or surface free energy for nanoscale clusters are inherently difficult. However, experimental nucleation rates for water at 300 K suggest that the surface free energy of droplets is higher than that of a planar surface by about 5 mN/m.^69,74–76^ In addition, experimental estimates of the nucleation barrier at 300 K exceed CNT predictions by approximately 5.9 *k*_B_ *T* at 300 K, based on eq (23) in ref 76, under the assumption that the difference in nucleation rates arises solely from differences in barrier height. To enable direct comparison with experimental observations, additional simulations were conducted to compute the nucleation free energy for clusters containing up to 150 molecules at *T* = 300 K. The results at supersaturations *S*_nucl_ = 3, 3.5, and 4 are presented in Figure 7a. For reference, predictions from CNT, based on eq 8, are also included. These supersaturation levels are comparable to those used in experiments, which ranged from 3.39 to 3.79.^75^ In all cases, CNT underestimates the nucleation barrier—by 5.6 *k*_B_ *T* at *S*_nucl_ = 3, 3.6 *k*_B_ *T* at *S*_nucl_ = 3.5, and 2.3 *k*_B_ *T* at *S*_nucl_ = 4. It is worth noting that, for water, the error introduced by CNT’s underestimation of the surface free energy for clusters is partially offset by another inherent limitation of the theory: its treatment of the monomer with a nonzero nucleation free energy.^22–24,54^ At higher supersaturations, smaller critical cluster sizes result in a better balance between these competing sources of error, leading to smaller deviations between CNT predictions and simulation data. These two opposing error sources may also help explain the observed temperature dependence of the deviation between CNT predictions and experimental results (see Figure 11 of ref 76), as the critical cluster size increases with temperature (see Figure 6 of ref 77). Ongoing simulation efforts aim to further investigate this hypothesis.

**Figure 7:**
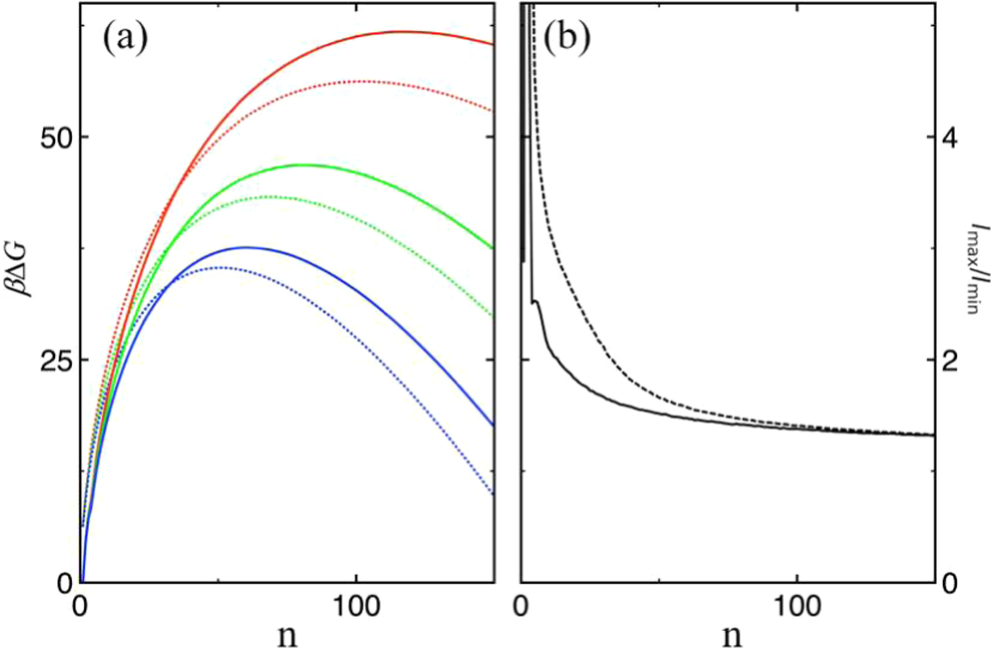
(a) Δ*G* in units of *k*_B_ *T* as a function of *n* obtained for TIP4P/2005 water at *S*_nucl_ = 3 (red), 3.5 (green), and 4 (blue) from the simulation (solid) and from CNT (dotted) using eq 8. (b) The average ratio of the largest to smallest principal moments of inertia as a function of cluster size for LJ (dashed) and water (solid).

The CNT errors observed from the experimental nucleation rates of water are remarkably small compared to those of other systems, such as argon.^76^ In addition to the error compensation mentioned above, water’s ability to form compact clusters through strong hydrogen bonding from the very onset may be another contributing factor. This is supported by the analysis of shape anisotropy, measured as the average ratio of the largest to the smallest principal moment of inertia as a function of cluster size (see Figure 7b). Compared to water, LJ forms fractal, nonspherical clusters over a much broader range of cluster sizes, which accounts for the more pronounced deviations from linearity observed in the Δ^2^ *G* results shown in Figure 3.

## 4 Conclusions

In summary, this work presents a new approach for calculating the surface tension of an infinite planar surface by leveraging the size-dependency of nucleation free energy, which can be efficiently obtained using the aggregation-volume-biased Monte Carlo method. For both Lennard–Jones and TIP4P/2005 water systems, the surface tension extrapolated from this cluster-based approach shows excellent agreement with values obtained from traditional bulk-phase methods. Comparison of the simulation results with classical nucleation theory highlights the necessity of incorporating the size dependence of surface tension for accurate modeling. For large clusters, this size dependence is well captured by the Tolman equation. For TIP4P/2005 water, the surface tension decreases notably with increasing cluster size, consistent with a negative Tolman length of −0.48 ± 0.02 Å. While there remains no consensus on the exact value or even the sign of the Tolman length, and direct experimental measurements are lacking, this result aligns with observed discrepancies between experimental nucleation rates and CNT predictions for water.

## Supplementary Material



## Notes

Tables containing the numerical values corresponding to the data presented in the figures (PDF)
