# Supplementary material for: Surface Tension of Infinitely Planar Surfaces from Nucleation Free Energies: A Comparison of Monte Carlo Calculations and Classical Theories
Source: J Chem Theory Comput. 2025 Aug 18;21(16):8051–9. doi: 10.1021/acs.jctc.5c01122 (PMC12392455; doi:10.1021/acs.jctc.5c01122)
Supplement: Supplementary file 1 [file ct5c01122_si_001.pdf]

Supporting Information for:

Surface Tension of Infinitely Planar Surfaces from Nucleation Free Energies: A Comparison of Monte Carlo Calculations and Classical Theories

Bin Chen*,[1] and Ngoc My Nhi Nguyen[2]

[1]Department of Chemistry, Louisiana State University,
Baton Rouge, Louisiana 70803-1804, USA
[2]Cain Department of Chemical Engineering, Louisiana State University,
Baton Rouge, Louisiana 70803-1804, USA

Table S1: Acceptance rates in percentage for different selection strategies as a function of cluster size.

Cluster Size	Strategy 1	Strategy 2	Strategy 3	Strategy 4
20	0.89	3.26	8.33	8.15
40	0.60	2.34	8.00	8.02
60	0.44	2.02	7.86	8.07
80	0.46	1.71	7.84	8.17
100	0.38	1.51	7.81	8.23
150	0.36	1.40	7.80	8.45
200	0.32	1.20	7.85	8.61
250	0.30	1.10	7.84	8.70
400	0.24	0.93	7.99	9.06
600	0.18	0.75	8.05	9.37
800	0.16	0.69	8.02	9.54
1000	0.16	0.62	8.24	9.74
2000	0.13	0.47	8.18	10.32
4000	0.11	0.47	8.27	10.53
8000	0.10	0.37	8.18	10.64

Strategy 1: random selection of both target and removal molecules.
Strategy 2: preferential selection of removal molecule only.
Strategy 3: preferential selection of both target and removal molecules with $\alpha = 0$.
Strategy 4: preferential selection of both target and removal molecules with $\alpha = 0.1\beta$.

Table S2: $\Delta^2 G$ in units of $k_\mathrm{B}T$ for different selection strategies as a function of cluster size.

Cluster Size	Strategy 1	Strategy 2	Strategy 3	Strategy 4
		TIP4P/2005 Water		
40	-0.2073 ± 0.0035	-0.2264 ± 0.0020	-0.2345 ± 0.0030	-0.2322 ± 0.0020
100	-1.9779 ± 0.0034	-1.9982 ± 0.0039	-2.0076 ± 0.0038	-2.0083 ± 0.0021
		Lennard-Jones (LJ)		
200	3.04627 ±0.00053			3.04508 ±0.00021
400	2.19160 ±0.00066			2.19028 ±0.00018
800	1.51257 ±0.00086			1.51067 ±0.00019

Strategy 1: random selection of both target and removal molecules.
Strategy 2: preferential selection of removal molecule only.
Strategy 3: preferential selection of both target and removal molecules with $\alpha = 0$.
Strategy 4: preferential selection of both target and removal molecules with $\alpha = 0.1\beta$.
Weighted linear fits to the $\Delta^2 G$ results obtained for LJ yield a slope of 9.088 ± 0.006 and an intercept of -1.098 ± 0.002 for Strategy 1, vs. a slope of 9.093 ± 0.002 and an intercept of -1.1012 ± 0.0006 for Strategy 4.

Table S3: Values of $\Delta^2 G = \Delta G(n+2) - \Delta G(n-2)$ in units of $k_{\mathrm{B}}T$ as a function of cluster size n for Lennard-Jones and TIP4P/2005 water models.

	Lennard-Jones		TIP4P/2005 Water	
n	$\Delta^2 G$	Uncertainty	$\Delta^2 G$	Uncertainty
20	7.25669	4.0×10^{-4}	1.5496	4.7×10^{-3}
40	5.92487	4.0×10^{-4}	-0.2322	2.0×10^{-3}
60			-1.0895	2.3×10^{-3}
80	4.51179	4.4×10^{-4}	-1.6279	3.4×10^{-3}
100			-2.0083	2.1×10^{-3}
150			-2.6241	5.7×10^{-3}
200	3.04508	2.1×10^{-4}	-3.0074	4.5×10^{-3}
250			-3.2867	5.9×10^{-3}
400	2.19028	1.8×10^{-4}	-3.7929	4.9×10^{-3}
600			-4.1660	5.8×10^{-3}
800	1.51067	1.9×10^{-4}	-4.4056	4.3×10^{-3}
1000			-4.5744	9.5×10^{-3}
2000	0.82324	5.6×10^{-4}	-5.0127	3.9×10^{-3}
4000	0.42667	2.4×10^{-4}	-5.3578	9.4×10^{-3}
8000	0.11238	3.9×10^{-4}	-5.6399	6.8×10^{-3}

Table S4: $\Delta^3 G/a(n,m)$ in units of $k_{\mathrm{B}}T$ (Part 1)

m	n	$\Delta_3 G/a(n,m)$	Uncertainty
20	40	8.766	0.0289
20	60	8.741	0.0187
20	80	8.724	0.014
20	100	8.707	0.0073
20	150	8.673	0.017
20	200	8.645	0.010
20	250	8.639	0.0097
20	400	8.600	0.0034
20	600	8.569	0.0094
20	800	8.558	0.0084
20	1000	8.547	0.013
20	2000	8.506	0.0093
20	4000	8.474	0.012
20	8000	8.460	0.0095
40	60	8.689	0.022
40	80	8.670	0.028
40	100	8.649	0.017
40	150	8.604	0.016
40	200	8.569	0.019
40	250	8.566	0.019
40	400	8.519	0.013
40	600	8.483	0.0096
40	800	8.472	0.0097
40	1000	8.461	0.016
40	2000	8.413	0.0083
40	4000	8.377	0.017
40	8000	8.364	0.013

Table S5: $\Delta^3 G/a(n,m)$ in units of $k_\mathrm{B}T$ (Part 2)

m	n	$\Delta^3 G/a(n,m)$	Uncertainty
60	80	8.640	0.053
60	100	8.612	0.031
60	150	8.557	0.026
60	200	8.516	0.027
60	250	8.519	0.029
60	400	8.467	0.016
60	600	8.428	0.016
60	800	8.417	0.014
60	1000	8.406	0.020
60	2000	8.355	0.012
60	4000	8.317	0.019
60	8000	8.305	0.012
80	100	8.572	0.061
80	150	8.513	0.048
80	200	8.468	0.040
80	250	8.481	0.029
80	400	8.425	0.022
80	600	8.384	0.026
80	800	8.375	0.022
80	1000	8.365	0.029
80	2000	8.311	0.012
80	4000	8.272	0.014
80	8000	8.262	0.0083
100	150	8.478	0.086
100	200	8.429	0.039
100	250	8.454	0.032
100	400	8.394	0.017
100	600	8.351	0.024
100	800	8.345	0.018
100	1000	8.335	0.031
100	2000	8.279	0.013
100	4000	8.239	0.020
100	8000	8.231	0.014

Table S6: $\Delta^3 G/a(n,m)$ in units of $k_\mathrm{B}T$ (Part 3)

m	n	$\Delta^3 G/a(n,m)$	Uncertainty
150	200	8.352	0.22
150	250	8.433	0.099
150	400	8.351	0.054
150	600	8.302	0.032
150	800	8.300	0.043
150	1000	8.291	0.035
150	2000	8.230	0.023
150	4000	8.187	0.027
150	8000	8.183	0.024
200	250	8.545	0.24
200	400	8.350	0.064
200	600	8.286	0.061
200	800	8.286	0.018
200	1000	8.277	0.062
200	2000	8.207	0.027
200	4000	8.161	0.042
200	8000	8.158	0.026
250	400	8.246	0.11
250	600	8.206	0.075
250	800	8.224	0.064
250	1000	8.221	0.080
250	2000	8.155	0.023
250	4000	8.112	0.026
250	8000	8.115	0.030

Table S7: $\Delta^3 G/a(n,m)$ in units of $k_\mathrm{B}T$ (Part 4)

m	n	$\Delta^3 G/a(n,m)$	Uncertainty
400	600	8.153	0.11
400	800	8.205	0.076
400	1000	8.204	0.075
400	2000	8.117	0.054
400	4000	8.069	0.055
400	8000	8.080	0.038
600	800	8.288	0.23
600	1000	8.251	0.12
600	2000	8.102	0.078
600	4000	8.043	0.084
600	8000	8.061	0.064
800	1000	8.200	0.49
800	2000	8.030	0.092
800	4000	7.984	0.11
800	8000	8.019	0.065
1000	2000	7.967	0.23
1000	4000	7.938	0.15
1000	8000	7.991	0.097
2000	4000	7.903	0.19
2000	8000	8.008	0.099
4000	8000	8.139	0.19

Table S8: Intercept and slope values in units of $k_\mathrm{B}T$ from weighted linear fits to $\Delta^3 G$ data performed over different cluster size ranges denoted $[n_1,\, n_2]$

n_1	n_2	Intercept	Unc. Int.	Slope	Unc. Slope
60	250 (Long)	7.930	0.056	2.735	0.267
80	250 (Long)	7.964	0.122	2.555	0.621
20	250 (Short)	8.058	0.031	2.216	0.115
40	250 (Short)	7.907	0.062	2.901	0.264
60	250 (Short)	7.813	0.132	3.360	0.625
80	250 (Short)	7.964	0.122	2.555	0.621
20	400	7.989	0.021	2.448	0.082
40	400	7.861	0.039	3.089	0.176
60	400	7.787	0.074	3.479	0.374
80	400	7.808	0.134	3.362	0.724
100	400	7.862	0.223	3.048	1.250
20	600	7.952	0.017	2.584	0.067
40	600	7.819	0.030	3.265	0.144
60	600	7.739	0.055	3.705	0.287
80	600	7.720	0.089	3.822	0.498
100	600	7.704	0.128	3.913	0.748
150	600	7.441	0.342	5.665	2.239
20	2000	7.887	0.009	2.836	0.041
40	2000	7.785	0.016	3.424	0.083
60	2000	7.738	0.025	3.717	0.144
80	2000	7.730	0.034	3.778	0.217
100	2000	7.724	0.044	3.825	0.289
150	2000	7.638	0.084	4.500	0.620
200	2000	7.560	0.110	5.143	0.848
250	2000	7.746	0.185	3.502	1.551
400	2000	7.589	0.317	5.043	2.874
20	8000	7.850	0.007	2.983	0.032
40	8000	7.762	0.011	3.531	0.064
60	8000	7.724	0.017	3.791	0.105
80	8000	7.716	0.022	3.857	0.151
100	8000	7.708	0.027	3.916	0.195
150	8000	7.669	0.044	4.269	0.353
200	8000	7.648	0.053	4.463	0.446
250	8000	7.754	0.078	3.388	0.717
400	8000	7.687	0.118	4.122	1.191
600	8000	7.580	0.228	5.286	2.499
800	8000	7.760	0.424	3.118	5.043

Table S9: Surface tension (mN/m), Tolman length (Å), and $\Delta\mu$ ($k_B T$) from weighted fits to $\Delta^2 G$ and $\Delta^3 G$ data over cluster size ranges $[n_1, n_2]$.

n_1	n_2	$\gamma_{\Delta^2 G}$	$\gamma_{\Delta^3 G}$	$\delta_{\Delta^2 G}$	$\delta_{\Delta^3 G}$	$\Delta\mu$
20	250	71.554 ± 0.559	71.454 ± 0.273	-0.260 ± 0.029	-0.265 ± 0.014	-1.695 ± 0.005
40	250	69.895 ± 0.573	70.112 ± 0.546	-0.365 ± 0.033	-0.354 ± 0.032	-1.681 ± 0.005
60	250	68.757 ± 0.997	69.276 ± 1.166	-0.448 ± 0.065	-0.415 ± 0.077	-1.672 ± 0.008
80	250	69.775 ± 2.747	70.291 ± 2.552	-0.369 ± 0.193	-0.337 ± 0.178	-1.679 ± 0.020
20	400	71.000 ± 0.481	70.836 ± 0.186	-0.289 ± 0.026	-0.295 ± 0.010	-1.689 ± 0.004
40	400	69.647 ± 0.365	69.705 ± 0.343	-0.381 ± 0.022	-0.379 ± 0.022	-1.679 ± 0.003
60	400	68.886 ± 0.545	69.044 ± 0.656	-0.440 ± 0.037	-0.431 ± 0.046	-1.673 ± 0.004
80	400	69.374 ± 1.175	69.233 ± 1.191	-0.399 ± 0.087	-0.415 ± 0.089	-1.676 ± 0.008
100	400	70.021 ± 1.902	69.716 ± 1.976	-0.345 ± 0.144	-0.374 ± 0.153	-1.681 ± 0.013
20	600	70.590 ± 0.447	70.512 ± 0.148	-0.311 ± 0.025	-0.313 ± 0.008	-1.685 ± 0.004
40	600	69.349 ± 0.310	69.332 ± 0.270	-0.399 ± 0.019	-0.402 ± 0.018	-1.676 ± 0.003
60	600	68.612 ± 0.402	68.625 ± 0.487	-0.459 ± 0.029	-0.461 ± 0.036	-1.671 ± 0.003
80	600	68.664 ± 0.801	68.450 ± 0.790	-0.455 ± 0.062	-0.477 ± 0.062	-1.671 ± 0.005
100	600	68.756 ± 1.187	68.317 ± 1.137	-0.446 ± 0.095	-0.490 ± 0.094	-1.672 ± 0.008
150	600	66.888 ± 3.092	65.984 ± 3.034	-0.639 ± 0.291	-0.734 ± 0.290	-1.662 ± 0.017
20	2000	69.820 ± 0.301	69.937 ± 0.084	-0.356 ± 0.019	-0.347 ± 0.005	-1.678 ± 0.002
40	2000	69.000 ± 0.185	69.027 ± 0.141	-0.423 ± 0.013	-0.424 ± 0.010	-1.673 ± 0.001
60	2000	68.520 ± 0.208	68.617 ± 0.218	-0.467 ± 0.017	-0.463 ± 0.018	-1.671 ± 0.001
80	2000	68.424 ± 0.325	68.539 ± 0.305	-0.477 ± 0.029	-0.471 ± 0.027	-1.670 ± 0.002
100	2000	68.383 ± 0.400	68.489 ± 0.388	-0.481 ± 0.036	-0.477 ± 0.036	-1.670 ± 0.002
150	2000	67.669 ± 0.666	67.726 ± 0.744	-0.568 ± 0.072	-0.568 ± 0.078	-1.667 ± 0.003
200	2000	67.162 ± 0.837	67.037 ± 0.978	-0.635 ± 0.096	-0.656 ± 0.108	-1.665 ± 0.004
250	2000	68.071 ± 1.052	68.684 ± 1.644	-0.507 ± 0.128	-0.436 ± 0.193	-1.668 ± 0.004
400	2000	66.621 ± 1.944	67.297 ± 2.812	-0.723 ± 0.265	-0.641 ± 0.365	-1.663 ± 0.008
20	8000	69.617 ± 0.261	69.609 ± 0.063	-0.368 ± 0.017	-0.366 ± 0.004	-1.676 ± 0.002
40	8000	68.894 ± 0.155	68.831 ± 0.100	-0.430 ± 0.011	-0.438 ± 0.008	-1.672 ± 0.001
60	8000	68.491 ± 0.166	68.494 ± 0.148	-0.470 ± 0.014	-0.473 ± 0.013	-1.670 ± 0.001
80	8000	68.422 ± 0.237	68.414 ± 0.199	-0.477 ± 0.022	-0.482 ± 0.019	-1.670 ± 0.001
100	8000	68.398 ± 0.278	68.351 ± 0.244	-0.480 ± 0.026	-0.490 ± 0.024	-1.670 ± 0.001
150	8000	68.072 ± 0.434	68.005 ± 0.393	-0.523 ± 0.050	-0.537 ± 0.044	-1.669 ± 0.002
200	8000	67.923 ± 0.540	67.814 ± 0.474	-0.544 ± 0.065	-0.563 ± 0.056	-1.668 ± 0.002
250	8000	68.556 ± 0.591	68.755 ± 0.693	-0.445 ± 0.078	-0.421 ± 0.089	-1.670 ± 0.002
400	8000	68.412 ± 0.951	68.161 ± 1.046	-0.469 ± 0.139	-0.517 ± 0.149	-1.670 ± 0.003
600	8000	68.720 ± 2.971	67.213 ± 2.022	-0.413 ± 0.480	-0.672 ± 0.318	-1.671 ± 0.005

Table S10: Data for Figure 7 (part 1)

n	CNT1	CNT2	CNT3	Sim1	Sim2	Sim3	$I_{\max}/I_{\min}$ (water)	$I_{\max}/I_{\min}$ (LJ)
1	6.61	6.46	6.32	0.00	0.00	0.00	2.88	
2	10.04	9.73	9.46	4.96	4.80	4.67	40.23	
3	12.74	12.28	11.88	7.73	7.42	7.16	4.97	46.13
4	15.03	14.41	13.88	9.21	8.74	8.34	2.51	6.16
5	17.05	16.28	15.61	11.72	11.11	10.57	2.53	4.58
6	18.86	17.94	17.13	14.09	13.32	12.65	2.52	4.03
7	20.52	19.44	18.50	16.09	15.17	14.37	2.43	3.69
8	22.04	20.81	19.74	17.85	16.77	15.84	2.31	3.51
9	23.46	22.08	20.88	19.47	18.24	17.17	2.19	3.35
10	24.79	23.25	21.92	21.01	19.62	18.42	2.11	3.20
11	26.04	24.35	22.88	22.45	20.91	19.57	2.07	3.11
12	27.22	25.37	23.77	23.82	22.12	20.65	2.03	3.03
13	28.34	26.33	24.60	25.12	23.27	21.67	2.01	2.95
14	29.40	27.24	25.37	26.37	24.37	22.63	1.97	2.87
15	30.40	28.09	26.09	27.56	25.40	23.53	1.94	2.81
16	31.37	28.90	26.76	28.70	26.39	24.39	1.92	2.76
17	32.29	29.67	27.40	29.80	27.33	25.19	1.89	2.70
18	33.17	30.39	27.99	30.85	28.23	25.96	1.87	2.64
19	34.01	31.08	28.55	31.86	29.08	26.68	1.84	2.60
20	34.82	31.74	29.07	32.82	29.89	27.36	1.82	2.55
21	35.60	32.37	29.56	33.76	30.67	28.00	1.80	2.48
22	36.35	32.96	30.02	34.66	31.42	28.62	1.78	2.45
23	37.07	33.53	30.46	35.53	32.14	29.20	1.76	2.39
24	37.77	34.07	30.87	36.38	32.83	29.76	1.75	2.34
25	38.44	34.59	31.25	37.19	33.49	30.28	1.72	2.31
26	39.09	35.08	31.61	37.97	34.12	30.78	1.72	2.26
27	39.71	35.55	31.95	38.74	34.73	31.26	1.70	2.22
28	40.32	36.00	32.26	39.47	35.31	31.70	1.69	2.17
29	40.90	36.43	32.56	40.17	35.86	32.12	1.68	2.14
30	41.47	36.84	32.83	40.86	36.39	32.52	1.66	2.11

Table S11: Data for Figure 7 (part 2)

n	CNT1	CNT2	CNT3	Sim1	Sim2	Sim3	$I_{\max}/I_{\min}$ (water)	$I_{\max}/I_{\min}$ (LJ)
31	42.01	37.23	33.09	41.53	36.91	32.90	1.65	2.06
32	42.54	37.61	33.33	42.18	37.41	33.27	1.64	2.03
33	43.05	37.96	33.56	42.82	37.89	33.61	1.63	2.00
34	43.55	38.31	33.77	43.43	38.35	33.94	1.62	1.97
35	44.03	38.63	33.96	44.03	38.79	34.25	1.61	1.94
36	44.49	38.94	34.14	44.60	39.21	34.54	1.60	1.91
37	44.94	39.24	34.30	45.16	39.61	34.80	1.59	1.89
38	45.38	39.52	34.45	45.70	40.00	35.06	1.59	1.86
39	45.80	39.79	34.58	46.23	40.37	35.29	1.58	1.83
40	46.21	40.05	34.71	46.74	40.73	35.52	1.58	1.82
41	46.61	40.29	34.82	47.24	41.07	35.73	1.57	1.80
42	47.00	40.52	34.91	47.73	41.41	35.93	1.56	1.78
43	47.37	40.74	35.00	48.20	41.72	36.11	1.55	1.76
44	47.73	40.95	35.08	48.66	42.03	36.29	1.55	1.74
45	48.09	41.15	35.14	49.11	42.32	36.45	1.54	1.73
46	48.43	41.33	35.19	49.54	42.60	36.59	1.54	1.71
47	48.76	41.51	35.24	49.96	42.87	36.73	1.53	1.70
48	49.08	41.68	35.27	50.38	43.14	36.86	1.53	1.68
49	49.39	41.83	35.29	50.78	43.38	36.97	1.52	1.68
50	49.69	41.98	35.30	51.17	43.62	37.08	1.52	1.66
51	49.98	42.12	35.31	51.56	43.85	37.17	1.51	1.65
52	50.26	42.25	35.30	51.92	44.06	37.25	1.51	1.64
53	50.54	42.37	35.29	52.29	44.27	37.33	1.51	1.63
54	50.80	42.48	35.27	52.64	44.47	37.39	1.49	1.63
55	51.06	42.58	35.24	52.98	44.66	37.45	1.49	1.61
56	51.31	42.67	35.20	53.31	44.84	37.49	1.49	1.60
57	51.55	42.76	35.15	53.64	45.00	37.53	1.49	1.59
58	51.78	42.84	35.10	53.95	45.16	37.55	1.48	1.59
59	52.01	42.91	35.03	54.26	45.31	37.57	1.47	1.58
60	52.22	42.97	34.96	54.55	45.46	37.58	1.48	1.57

Table S12: Data for Figure 7 (part 3)

n	CNT1	CNT2	CNT3	Sim1	Sim2	Sim3	$I_{\max}/I_{\min}$ (water)	$I_{\max}/I_{\min}$ (LJ)
61	52.43	43.03	34.89	54.83	45.58	37.57	1.47	1.56
62	52.64	43.08	34.80	55.11	45.70	37.56	1.47	1.56
63	52.83	43.12	34.71	55.37	45.82	37.54	1.46	1.55
64	53.02	43.16	34.61	55.64	45.93	37.52	1.46	1.54
65	53.21	43.19	34.51	55.90	46.03	37.49	1.46	1.54
66	53.38	43.21	34.40	56.15	46.13	37.45	1.45	1.53
67	53.55	43.22	34.28	56.40	46.23	37.41	1.45	1.53
68	53.72	43.23	34.15	56.63	46.31	37.36	1.45	1.52
69	53.87	43.24	34.02	56.86	46.38	37.30	1.45	1.52
70	54.02	43.23	33.89	57.09	46.45	37.24	1.44	1.51
71	54.17	43.22	33.74	57.31	46.52	37.17	1.44	1.50
72	54.31	43.21	33.60	57.52	46.58	37.10	1.44	1.50
73	54.44	43.19	33.44	57.74	46.64	37.02	1.43	1.50
74	54.57	43.16	33.28	57.94	46.68	36.94	1.43	1.49
75	54.69	43.13	33.12	58.13	46.73	36.84	1.43	1.49
76	54.81	43.10	32.95	58.32	46.76	36.74	1.42	1.49
77	54.92	43.05	32.77	58.50	46.78	36.64	1.42	1.48
78	55.03	43.01	32.59	58.68	46.81	36.53	1.43	1.48
79	55.13	42.95	32.40	58.84	46.82	36.40	1.42	1.47
80	55.23	42.90	32.21	59.00	46.82	36.28	1.42	1.47
81	55.32	42.83	32.02	59.15	46.82	36.14	1.42	1.47
82	55.41	42.77	31.82	59.31	46.82	36.00	1.41	1.46
83	55.49	42.69	31.61	59.46	46.81	35.87	1.41	1.46
84	55.56	42.62	31.40	59.60	46.80	35.72	1.41	1.46
85	55.64	42.53	31.18	59.73	46.78	35.57	1.40	1.45
86	55.71	42.45	30.96	59.87	46.76	35.41	1.41	1.45
87	55.77	42.36	30.74	60.00	46.74	35.26	1.40	1.44
88	55.83	42.26	30.51	60.13	46.71	35.10	1.40	1.44
89	55.88	42.16	30.28	60.24	46.68	34.93	1.40	1.44
90	55.93	42.06	30.04	60.36	46.64	34.75	1.39	1.44

Table S13: Data for Figure 7 (part 4)

n	CNT1	CNT2	CNT3	Sim1	Sim2	Sim3	$I_{\mathrm{max}}/I_{\mathrm{min}}$ (water)	$I_{\mathrm{max}}/I_{\mathrm{min}}$ (LJ)
91	55.98	41.95	29.80	60.47	46.59	34.57	1.39	1.43
92	56.02	41.84	29.55	60.57	46.54	34.39	1.39	1.43
93	56.06	41.72	29.30	60.67	46.49	34.20	1.39	1.43
94	56.09	41.60	29.05	60.76	46.43	34.01	1.38	1.42
95	56.12	41.48	28.79	60.85	46.36	33.81	1.39	1.42
96	56.15	41.35	28.53	60.94	46.30	33.61	1.38	1.42
97	56.17	41.22	28.26	61.02	46.22	33.41	1.38	1.42
98	56.19	41.08	27.99	61.10	46.15	33.20	1.38	1.41
99	56.20	40.94	27.72	61.18	46.07	32.99	1.38	1.41
100	56.21	40.80	27.44	61.25	45.99	32.77	1.38	1.41
101	56.22	40.65	27.16	61.32	45.91	32.55	1.38	1.41
102	56.22	40.50	26.88	61.38	45.81	32.32	1.37	1.41
103	56.22	40.34	26.59	61.43	45.71	32.09	1.37	1.40
104	56.22	40.18	26.30	61.48	45.61	31.85	1.37	1.40
105	56.21	40.02	26.00	61.53	45.50	31.61	1.37	1.40
106	56.20	39.86	25.70	61.57	45.39	31.37	1.37	1.39
107	56.18	39.69	25.40	61.61	45.27	31.12	1.37	1.39
108	56.17	39.52	25.10	61.64	45.15	30.86	1.37	1.39
109	56.14	39.34	24.79	61.67	45.02	30.60	1.36	1.39
110	56.12	39.16	24.47	61.70	44.90	30.34	1.36	1.39
111	56.09	38.98	24.16	61.73	44.77	30.08	1.36	1.38
112	56.06	38.80	23.84	61.75	44.64	29.82	1.36	1.38
113	56.03	38.61	23.52	61.77	44.51	29.55	1.36	1.38
114	55.99	38.42	23.19	61.79	44.37	29.28	1.35	1.38
115	55.95	38.22	22.87	61.79	44.22	29.00	1.35	1.37
116	55.91	38.02	22.53	61.79	44.07	28.71	1.35	1.38
117	55.86	37.82	22.20	61.79	43.91	28.42	1.35	1.37
118	55.81	37.62	21.86	61.79	43.75	28.13	1.35	1.37
119	55.76	37.41	21.52	61.79	43.60	27.84	1.35	1.37
120	55.70	37.20	21.18	61.78	43.44	27.55	1.35	1.37

Table S14: Data for Figure 7 (part 5)

n	CNT1	CNT2	CNT3	Sim1	Sim2	Sim3	$I_{\max}/I_{\min}$ (water)	$I_{\max}/I_{\min}$ (LJ)
121	55.64	36.99	20.83	61.77	43.28	27.25	1.35	1.37
122	55.58	36.78	20.49	61.76	43.11	26.95	1.35	1.36
123	55.52	36.56	20.13	61.75	42.94	26.65	1.34	1.37
124	55.45	36.34	19.78	61.73	42.77	26.35	1.34	1.36
125	55.38	36.11	19.42	61.71	42.60	26.04	1.34	1.36
126	55.31	35.89	19.06	61.69	42.42	25.73	1.34	1.36
127	55.24	35.66	18.70	61.67	42.24	25.42	1.34	1.36
128	55.16	35.43	18.34	61.64	42.06	25.10	1.34	1.35
129	55.08	35.19	17.97	61.61	41.88	24.79	1.34	1.35
130	55.00	34.96	17.60	61.57	41.69	24.46	1.34	1.35
131	54.91	34.72	17.22	61.53	41.49	24.13	1.34	1.35
132	54.82	34.47	16.85	61.49	41.30	23.80	1.34	1.35
133	54.73	34.23	16.47	61.45	41.10	23.48	1.34	1.35
134	54.64	33.98	16.09	61.40	40.90	23.14	1.34	1.35
135	54.54	33.73	15.71	61.35	40.70	22.80	1.34	1.35
136	54.44	33.48	15.32	61.30	40.49	22.47	1.33	1.34
137	54.34	33.23	14.93	61.25	40.28	22.12	1.33	1.34
138	54.24	32.97	14.54	61.19	40.07	21.78	1.33	1.34
139	54.14	32.71	14.15	61.12	39.85	21.42	1.33	1.34
140	54.03	32.45	13.75	61.06	39.63	21.07	1.33	1.34
141	53.92	32.18	13.35	60.99	39.41	20.72	1.33	1.34
142	53.81	31.92	12.95	60.92	39.19	20.36	1.33	1.34
143	53.69	31.65	12.55	60.85	38.96	20.00	1.32	1.34
144	53.57	31.38	12.15	60.79	38.74	19.65	1.32	1.33
145	53.45	31.10	11.74	60.72	38.52	19.29	1.32	1.33
146	53.33	30.83	11.33	60.65	38.29	18.93	1.32	1.33
147	53.21	30.55	10.92	60.57	38.06	18.57	1.32	1.33
148	53.08	30.27	10.51	60.50	37.83	18.21	1.32	1.33
149	52.95	29.99	10.09	60.42	37.60	17.84	1.32	1.33
150	52.82	29.70	9.67	60.33	37.36	17.47	1.32	1.33
